# Non-contiguous finished genome sequence of plant-growth promoting *Serratia proteamaculans* S4

**DOI:** 10.4056/sigs.4027757

**Published:** 2013-07-30

**Authors:** Saraswoti Neupane, Lynne A. Goodwin, Nils Högberg, Nikos C. Kyrpides, Sadhna Alström, David Bruce, Beverly Quintana, Christine Munk, Hajnalka Daligault, Hazuki Teshima, Karen Davenport, Krista Reitenga, Lance Green, Patrick Chain, Tracy Erkkila, Wei Gu, Xiaojing Zhang, Yan Xu, Yulia Kunde, Olga Chertkov, James Han, Cliff Han, John C. Detter, Natalia Ivanova, Amrita Pati, Amy Chen, Ernest Szeto, Kostas Mavromatis, Marcel Huntemann, Matt Nolan, Sam Pitluck, Shweta Deshpande, Victor Markowitz, Ioanna Pagani, Hans-Peter Klenk, Tanja Woyke, Roger D. Finlay

**Affiliations:** 1 Uppsala BioCenter, Department of Forest Mycology and Plant Pathology, Swedish University of Agricultural Sciences, Uppsala, Sweden; 2 Los Alamos National Laboratory, Bioscience Division, Los Alamos, New Mexico, USA,; 3 DOE Joint Genome Institute, Walnut Creek, California, USA,; 4 Leibniz Institute DSMZ – German Collection of Microorganisms and Cell Cultures, Braunschweig, Germany

**Keywords:** Facultative aerobe, gram-negative, motile, non-sporulating, mesophilic, chemoorganotrophic, agriculture

## Abstract

*Serratia proteamaculans* S4 (previously *Serratia sp.* S4), isolated from the rhizosphere of wild *Equisetum* sp., has the ability to stimulate plant growth and to suppress the growth of several soil-borne fungal pathogens of economically important crops. Here we present the non-contiguous, finished genome sequence of *S. proteamaculans* S4, which consists of a 5,324,944 bp circular chromosome and a 129,797 bp circular plasmid. The chromosome contains 5,008 predicted genes while the plasmid comprises 134 predicted genes. In total, 4,993 genes are assigned as protein-coding genes. The genome consists of 22 rRNA genes, 82 tRNA genes and 58 pseudogenes. This genome is a part of the project “Genomics of four rapeseed plant growth-promoting bacteria with antagonistic effect on plant pathogens” awarded through the 2010 DOE-JGI’s Community Sequencing Program.

## Introduction

The genus *Serratia* is a diverse and widely dispersed group of *Gammaproteobacteria* [1,2]. Some of these have beneficial effects on ecologically and economically important plants [3-4] and others are known as opportunistic pathogens of humans and other organisms [1]. Plant-associated *Serratia* spp. are of considerable agricultural interest and several strains of *S. plymuthica* have recently been studied in relation to their possible use as biocontrol agents in agriculture [3-4].

*Serratia proteamaculans* S4 (previously *Serratia sp.* S4) was isolated from the rhizosphere of naturally growing *Equisetum* plants in 1980 from Uppsala, Sweden. The bacterium is able to enhance the growth of rapeseed plants and inhibit the growth of different fungal pathogens such as *Verticillium dahliae,* and *Rhizoctonia solani*. Sequencing the *S. proteamaculans* S4 genome will therefore assist in the identification of genetic traits underlying its potential and its beneficial effects on plant growth. Here we present the non-contiguous finished genome sequence of *S. proteamaculans* S4.

### Classification and features

A representative 16S rRNA gene sequence of *S. proteamaculans* S4 was subjected to comparison with the most recently released databases in GenBank. The NCBI BLAST [5] tool was used under the default settings (i.e. by considering only the high-scoring segment pairs (HSP’s) from the best 250 hits). The most frequently matching genus was *Serratia* (almost 50% of total matches). When considering high score, coverage and identity – *S. proteamaculans* 568 was the first match with 100% identity and 100% coverage. Other *Serratia* species with maximum identity were other *S. proteamaculans* strains (10%) with maximum identity 99%, *S. fonticola* (2%) with maximum identity 98%, *S. grimesii* (3.2%) with maximum identity 99%, *S. liquefaciens* (4.4%) with maximum identity 99%, *S. plymuthica* (3.2%) maximum identity 98-99% and unclassified *Serratia sp.* (22%) with maximum identity 98-99%. Remaining matches were with *Rahnella* sp. (2%) with maximum identity 98-99% and other uncultured bacterial clones (40%) with maximum identity 98-99%.

**Figure 1:**
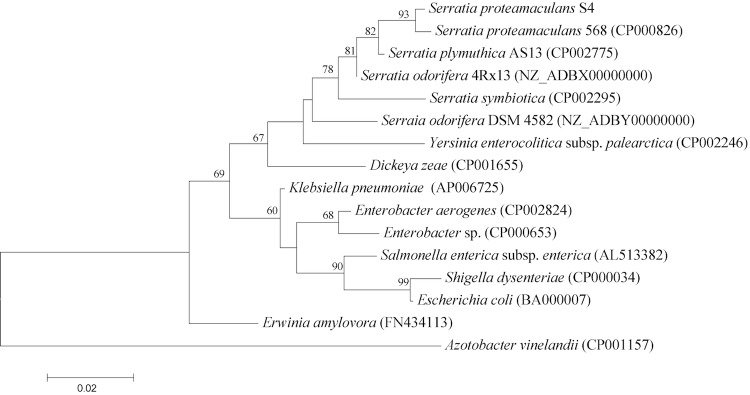
Phylogenetic tree highlighting the position of *S. proteamaculans* S4 in relation to other type and non-type strains within the family *Enterobacteriaceae*. The tree is based on 1,489 characters of the 16S rRNA gene sequence aligned in ClustalW2 [6] under the default settings. The tree was constructed using MEGA5 software [7] under the Maximum likelihood criterion and the tree was rooted with *Azotobacter vinelandii* (a member of the family *Pseudomonadaceae*). The branches are scaled according to the expected number of substitutions per site. The numbers above the branches are support values from 1,000 bootstrap replicates if larger than 60% [8]. All lineages with genome sequences are registered in GOLD [9].

Figure 1 shows the phylogenetic proximity of *S. proteamaculans* S4 to *S. proteamaculans* 568 (CP000826) as well as its distinct separation from other members of the *Enterobacteriaceae*. Its phylogenetic relationship was further confirmed by digital DNA-DNA hybridization [10] values above 70% with the genome sequence of the *S. proteamaculans* 568 using the GGDC web-server [11].

**Figure 2:**
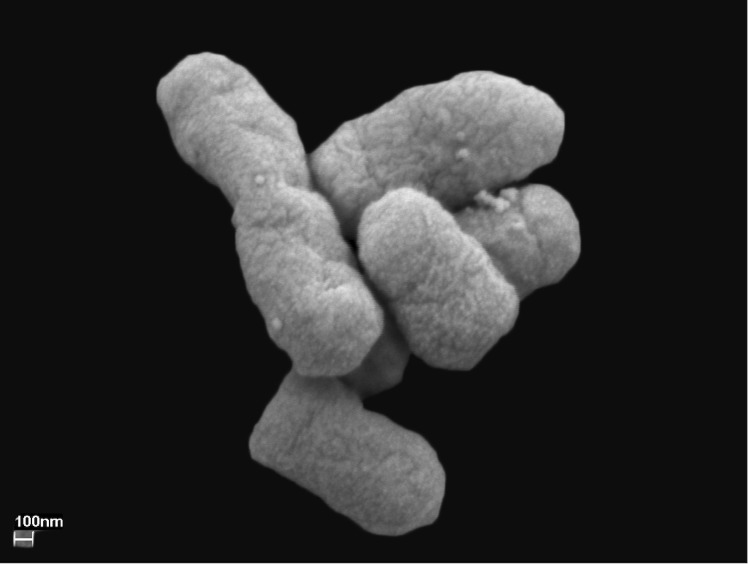
Scanning electron micrograph of *S. proteamaculans* S4

*Serratia proteamaculans* S4, a Gram-negative, rod shaped, non-sporulating and motile bacterium measuring 1-2 µm in length and 0.5-0.7 µm in width [Figure 2], was isolated from *Equisetum* roots. The bacterium is a pale yellow colored, facultative aerobe and easily grows on a broad spectrum of organic compounds including carbon sources such as glucose, sucrose, succinate, mannitol, inositol, sorbitol, arabinose, trehalose, and melibiose. The optimal temperature for its growth is 28 °C and it can grow in the pH range 4 – 10 [Table 1].

**Table 1:** Classification and general features of *S. proteamaculans* S4 according to the MIGS recommendations [12]

**MIGS ID**	**Property**	**Term**	**Evidence code**^a^
	Current classification	Domain *Bacteria*	TAS [13]
		Phylum *Proteobacteria*	TAS [14]
		Class *Gammaproteobacteria*	TAS [15,16]
		Order *Enterobacteriales*	TAS [17]
		Family *Enterobacteriaceae*	TAS [18-20]
		Genus *Serratia*	TAS [18,21,22]
		Species *Serratia proteamaculans*	TAS [18,23]
		Strain S4	IDA
	Gram stain	Negative	IDA
	Cell shape	Rod	IDA
	Motility	Motile	IDA
	Sporulation	Non-sporulating	IDA
	Temperature range	4 – 40 °C	IDA
	Optimum temperature	28 °C	IDA
	Carbon source	Glucose, sucrose, succinate, mannitol, arabinose, sorbitol, inositol	IDA
	Energy source	Chemoorganotrophic	IDA
MIGS-6	Habitat	Wild *Equisetum* rhizosphere	IDA
MIGS-6.3	Salinity	Medium	IDA
MIGS-22	Oxygen	Facultative	IDA
MIGS-15	Biotic relationship	Plant associated	IDA
MIGS-14	Pathogenicity Biosafety level	None 1	NAS TAS [24]
MIGS-4	Geographic location	Uppsala, Sweden	NAS
MIGS-5	Sample collection time	1980	NAS
MIGS-4.1	Latitude –	59	NAS
MIGS-4.2	Longitude	17	NAS
MIGS-4.3	Depth	0.1 m	NAS
MIGS-4.4	Altitude	58 - 63 m	NAS

a) Evidence codes - IDA: Inferred from Direct Assay; TAS: Traceable Author Statement (i.e., a direct report exists in the literature); NAS: Non-traceable Author Statement (i.e., not directly observed for the living, isolated sample, but based on a generally accepted property for the species, or anecdotal evidence). These evidence codes are from the Gene Ontology project [25]. If the evidence code is IDA, then the property should have been directly observed, for the purpose of this specific publication, for a live isolate by one of the authors, or an expert or reputable institution mentioned in the acknowledgements.

### Genome sequencing information

*Serratia proteamaculans* S4 was selected for sequencing because of its biological control potential and plant growth enhancing activity in rapeseed crops. The genome sequence is deposited in the Genomes On Line Databases [9]. Sequencing, finishing and annotation were performed by the DOE Joint Genome Institute (JGI). A summary of the project information is shown in Table 2 together with associated MIGS identifiers [12].

**Table 2:** Genome sequencing project information

**MIGS ID**	**Property**	**Term**
MIGS-31	Finishing quality	Non-contiguous Finished
MIGS-28	Libraries used	Three libraries: one 454 standard library, one paired end 454 library (10 kb insert size) and one Illumina library
MIGS-29	Sequencing platforms	Illumina GAii, 454 GS FLX Titanium
MIGS-31.2	Fold coverage	767.4 × Illumina, 8.7 × pyrosequencing
MIGS-30	Assemblers	Velvet version 1.1.05, Newbler version 2.6, phrap version SPS – 4.24
MIGS-32	Gene calling method	Prodigal (1.4), GenePRIMP
	NCBI project ID	61833
	NCBI taxon ID	768491
	IMG object ID	2508501071
	GOLD ID	Gi08429
	Project relevance	Biocontrol, Agriculture

### Growth conditions and DNA isolation

*Serratia proteamaculans* S4 was grown on Luria Broth (LB) medium for 12 hours at 28 °C. The DNA was extracted from the cells by using a standard CTAB protocol for bacterial genomic DNA isolation, which is available at JGI [26].

### Genome sequencing and assembly

The draft genome of *S. proteamaculans* S4 was generated using a combination of Illumina and 454 sequencing platforms. The details of library construction and sequencing are available at the JGI [26]. The sequence data generated from Illumina GAii (4,232 Mb) were assembled with Velvet [27] and the consensus sequence was computationally shredded into 1.5 kb overlapping fake reads. The sequencing data generated from 454 pyrosequencing (89.5 Mb) were assembled with Newbler and consensus sequences were computationally shredded into 2 kb overlapping fake reads. The initial draft assembly contained 50 contigs in 2 scaffolds. The 454 Newbler consensus reads, the Illumina Velvet consensus reads and the read pairs in the 454 paired end library were integrated using parallel Phrap [28,29]. The software, Consed [30] was used for the subsequent finishing process. The software Polisher [31] was used to correct the base errors and increase the consensus quality. Possible mis-assemblies were corrected with gapResolution ([26], unpublished), Dupfinisher [32] or by sequencing cloned bridging PCR fragments with subcloning. The gaps between contigs were closed by editing in the software Consed [30], by PCR and by Bubble PCR (J.-F. Chang, unpublished) primer walks. A total of 95 additional reactions was necessary to close gaps and to raise the quality of the finished sequence. The final assembly is based on 47 Mb of 454 draft data which provides an average 8.7 × coverage of the genome and 4,143.8 Mb of Illumina draft data, which provides an average 767.4 × coverage of the genome.

### Genome annotation

The *S. proteamaculans* S4 genes were identified using Prodigal [33] as part of the DOE-JGI annotation pipeline [34] followed by a round of manual curation using the JGI GenePRIMP pipeline [35]. The predicted CDSs were translated and used to search the National Center for Biotechnology Information (NCBI) non-redundant database, UniProt, TIGRFam, Pfam, PRIAM, KEGG, COG, and InterPro databases. These data sources were combined to assert a product description for each predicted protein. Non-coding genes and miscellaneous features were predicted using tRNAscan-SE [36], RNAmmer [37], Rfam [38], TMHMM [39], and signalP [40]. Additional gene prediction analysis and manual functional annotation was performed within the Integral Microbial Genomics-Expert Review (IMG-ER) [41] platform developed by the Joint Genome Institute, Walnut Creek, CA, USA.

## Genome properties

The genome includes a circular chromosome of 5,324,944 bp (55% GC content) along with a circular plasmid of 129,797 bp (50% GC content). The chromosome comprises 5,008 predicted genes while the plasmid comprises 137 predicted genes. In total 4,993 genes are assigned as protein-coding genes. About 85% of the protein-coding genes were assigned to a putative function with the remaining annotated as hypothetical proteins. The genome consists of 22 rRNA genes, 82 tRNA genes and 58 pseudogenes. The properties and the statistics of the genome are summarized in Tables 3 and 4 and Figures 3a and 3b.

**Table 3:** Genome statistics

**Attribute**	Value	% of total^a^
Genome size (bp)	5,454,741	100.00
DNA coding region (bp)	4,825,361	88.46
DNA G+C content (bp)	2,999,404	54.99
Total genes	5,142	100.00
RNA genes	149	2.90
rRNA operons	7	
Protein-coding genes	4,993	97.10
Pseudogenes	58	1.13
Genes in paralog clusters	2,759	53.66
Genes assigned to COGs	4,247	82.59
Genes with signal peptides	1,154	22.44
Genes with transmembrane helices	1,236	24.04

a) The total is based on either the size of the genome in base pairs or the total number of protein coding genes in the annotated genome.

**Table 4:** Number of genes associated with the 25 general COG functional categories

**Code**	**Value**	**% of total**^a^	**Description**
J	201	4.18	Translation
A	1	0.02	RNA processing and modification
K	452	9.41	Transcription
L	158	3.29	Replication, recombination and repair
B	1	0.02	Chromatin structure and dynamics
D	37	0.77	Cell cycle control, mitosis and meiosis
Y	0	0.00	Nuclear structure
V	57	1.19	Defense mechanisms
T	198	4.12	Signal transduction mechanisms
M	256	5.33	Cell wall/membrane biogenesis
N	142	2.96	Cell motility
Z	0	0.00	Cytoskeleton
W	0	0.00	Extracellular structures
U	166	3.46	Intracellular trafficking and secretion
O	153	3.18	Posttranslational modification, protein turnover, chaperones
C	275	5.72	Energy production and conversion
G	427	8.89	Carbohydrate transport and metabolism
E	487	10.14	Amino acid transport and metabolism
F	109	2.27	Nucleotide transport and metabolism
H	179	3.73	Coenzyme transport and metabolism
I	139	2.89	Lipid transport and metabolism
P	287	5.97	Inorganic ion transport and metabolism
Q	122	2.54	Secondary metabolite biosynthesis, transport and catabolism
R	549	11.43	General function prediction only
S	408	8.49	Function unknown
-	895	17.41	Not in COGs

a) The total is based on the total number of protein coding genes in the annotated genome.

**Figure 3a:**
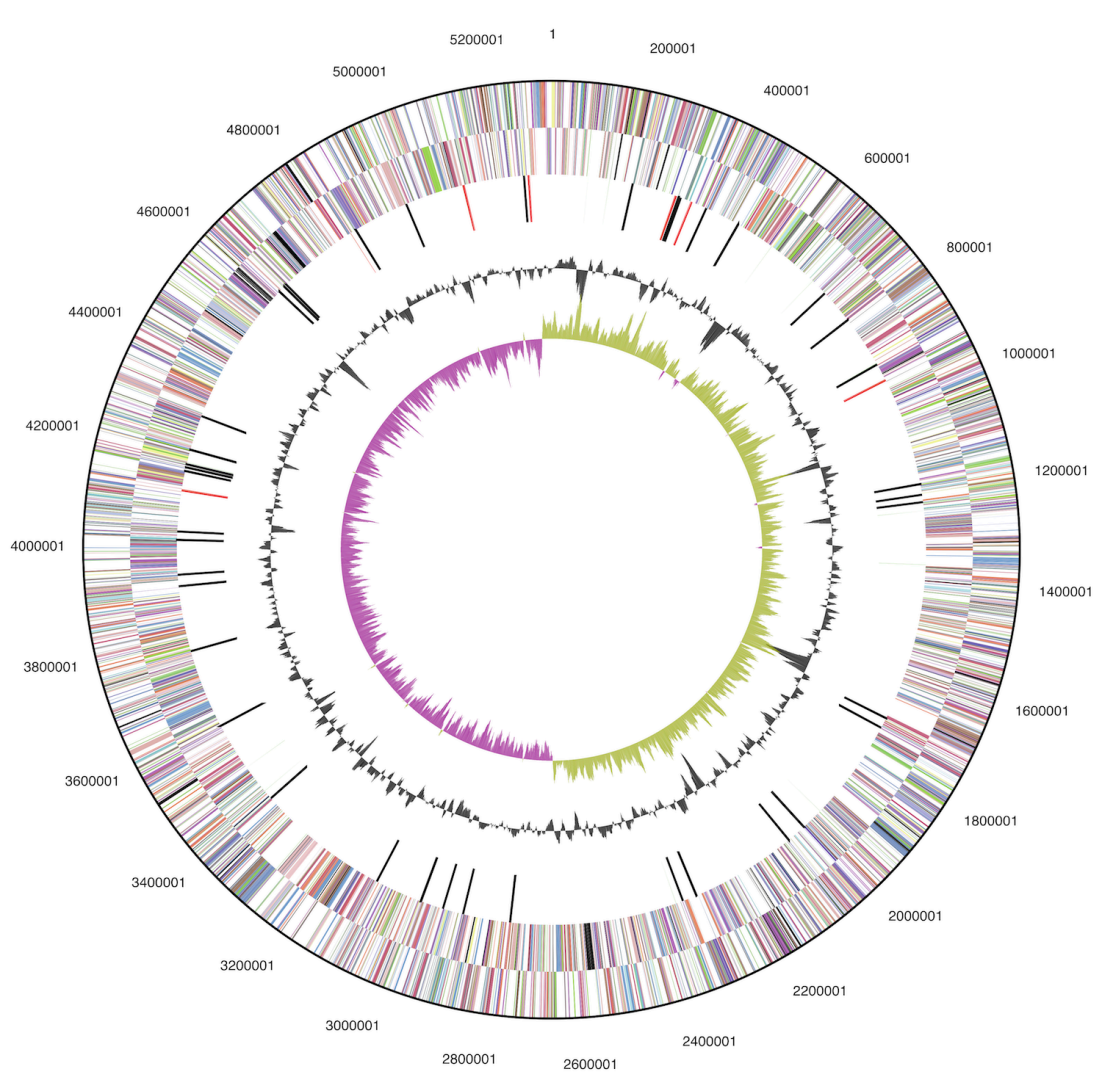
Graphical circular map of the chromosome. From outside to the center: Genes on forward strand (color by COG categories), Genes on reverse strand (color by COG categories), RNA genes (tRNAs blue, rRNAs red, other RNAs black), GC content, GC skew.

**Figure 3b:**
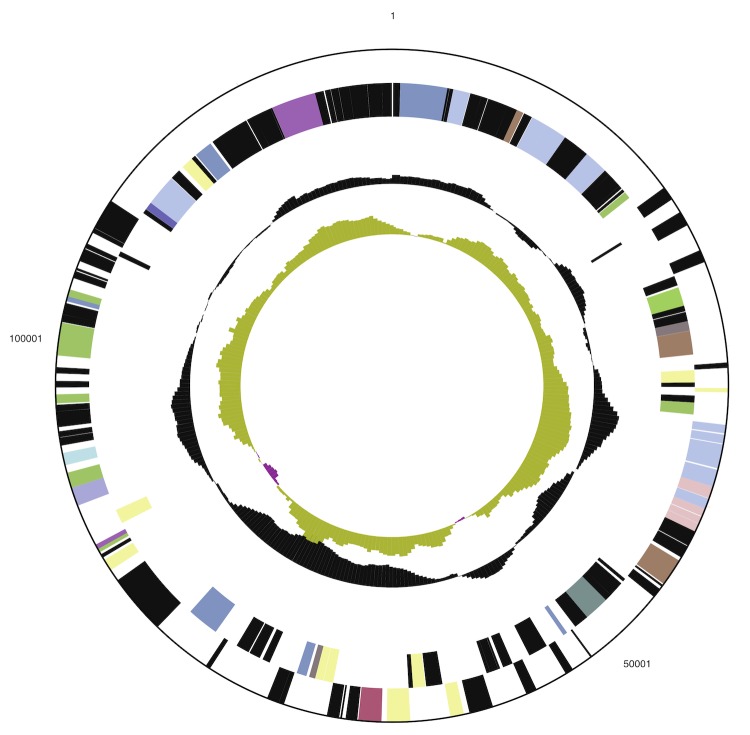
Graphical circular map of the plasmid. From outside to the center: Genes on forward strand (color by COG categories), Genes on reverse strand (color by COG categories), RNA genes (tRNAs blue, rRNAs red, other RNAs black), GC content, GC skew.

The genome contains genes arranged in several gene clusters encoding secondary metabolites such as siderophores (enterobactin and aerobactin) and antibiotics (pyrrolnitrin). These compounds can contribute indirectly to plant growth enhancement by suppressing growth of pathogens. The genome also includes genes for the production of plant growth hormones such as indole-3-acetic acid (IAA), which can be directly involved in plant growth. Further studies of the biochemical properties of additional secondary metabolites and regulation of their production using functional genomics will elucidate the detailed mechanisms underlying plant growth promotion by *S. proteamaculans* S4.
